# A simple and practical score model for predicting the mortality of severe fever with thrombocytopenia syndrome patients

**DOI:** 10.1097/MD.0000000000005708

**Published:** 2016-12-30

**Authors:** Shue Xiong, Wenjing Zhang, Mingyue Li, Yan Xiong, Mengmeng Li, Hua Wang, Dongliang Yang, Cheng Peng, Xin Zheng

**Affiliations:** Department of Infectious Diseases, Institute of Infection and Immunology, Union Hospital, Tongji Medical College, Huazhong University of Science and Technology, Wuhan, China.

**Keywords:** risk factor, score model, severe fever with thrombocytopenia syndrome

## Abstract

Severe fever with thrombocytopenia syndrome (SFTS) is an emerging disease with a high fatality rate. The risk factors for death are not clearly identified, and there is no clinical score model to predict the prognosis. We retrospectively collected the clinical information of clinical symptoms and laboratory parameters of SFTS patients on admission. After analyzing the clinical characteristics of 179 SFTS patients, we found that an elevated level of neurologic symptoms, respiratory symptoms, viral load, and a lower level of monocyte percentage were the critical risk factors for mortality. We used the 4 variables to assemble a score formula named the SFTS index [SFTSI = 5 × Neurologic symptoms-level + 4 × Respiratory symptoms-level + 3 × LG10 Viral load – 2 × LN Monocyte% – 7]. The AURC of this model was 0.964, which was higher than the AURC 0.913 of the viral load especially among the patients with higher viral loads (0.936 vs 0.821). We identified that the neurologic symptoms, respiratory symptoms, viral load, and monocyte percentage were the critical risk factors for SFTS mortality. The clinical score model of SFTSI provides a practical method for clinicians to stratify patients with SFTS and to adopt prompt effective treatment strategies.

## Abstract 2

Supplemental Digital Content is available in the text

## 1 Introduction

Severe fever with thrombocytopenia syndrome (SFTS) is an emerging disease caused by a novel bunyavirus and was first reported in China 2011 with an estimated high case-fatality rate of 12% to 30%.^[1]^ To date, the disease has been reported in mainland China, Japan, Korea,^[2,3]^ and the United States.^[4]^ The wide distribution and high case-fatality rate have made this new infectious disease a significant public health problem worldwide. In China, SFTS presents the epidemic characteristic of local prevalence, and most patients live in undeveloped areas, which adds to the burden of primary care physicians to engage these patients. As a new acute infectious disease, the clinical situation changes quickly, and as observed in clinical work, the referral of serious SFTS patients to the intensive care unit (ICU) in time was associated with an increased survival rate. Therefore, it is important for physicians, especially primary care clinicians, to recognize patients who are experiencing severe situations with probably the worst prognosis as early as possible.

In critically ill SFTS patients, the clinical conditions of serious patients could deteriorate rapidly and end in multiorgan failure (MOF) and death.^[1]^ Some previously published works assessed risk factors for death and severity from different aspects among SFTS patients. However, there were still no consistent conclusions derived, and the risk factors for death remained to be determined. To the best of our knowledge, there is not any study focusing on the prognostic score system of SFTS patients yet published. An early and accurate predictive model for outcomes of SFTS patients could help clinicians make a better decision and improve the efficiency of the treatment.

In this study, we summarize the laboratory parameters, clinical features, outcomes, and identify the 4 critical risk factors associated with fatal outcomes among SFTS patients in dozens of counties of the Hubei and Henan provinces, China from March to December 2015. The study was carried out by multiple regression analyses to construct a simple and practical scoring system that combines clinical symptoms and laboratory parameters for the prediction of SFTS patients’ mortality.

## 2 Methods

### 2.1 Surveillance system and case definition

A total of 179 patients who were admitted to Union Hospital, Wuhan, between March and November 2015 were enrolled in our study. We retrospectively collected the clinical information of clinical symptoms and laboratory parameters on admission and the mean duration day of disease course was 8.24 ± 2.57. Patients were excluded if they were coinfected by other viruses or had a history of other serious chronic diseases. SFTS patients were diagnosed according to the presence of an acute fever (temperature of 38°C or higher) and thrombocytopenia (platelet count <100 × 10^9^/L) and their laboratory results confirmed an SFTS virus (SFTSV) infection by real-time PCR. We followed up with the serious patients who stopped therapy to determine the final disease outcome. The research protocol was approved by the Ethics Committee of Tongji Medical College of Huazhong University of Science and Technology.

### 2.2 Clinical data

We collected all of following the data from each subject: demographic factors, comorbidity conditions, physical examination, and laboratory findings. The laboratory findings were analyzed within 24 hours of admission.

### 2.3 Statistical analysis

Statistical analyses were performed using the unpaired *t* test or Mann–Whitney *U* test (for continuous variables) to test the relationships between the fatal and nonfatal cases. The majority of the continuous variables were analyzed after transformation to ranked data or logarithmic form. Comparisons of the clinical parameters between groups were carried out by the Pearson *χ*^2^ or Fisher exact test in tables. We used the Pearson test to assess the correlation between variables. Risk factors were calculated by univariate and multivariate logistic regression analyses. The score methods of respiratory and neurologic symptoms are shown in Supplementary Table 1. Multiple linear regression analyses were used to assess the contribution of the clinical features to the mortality of SFTS patients. The predictive value of the model was evaluated by the ROC curve (AURC). The cut-off values were chosen to produce a simple and reliable model. The computations were carried out with statistical software package SPSS 21.0 (SPSS, an IBM Company, Armonk, NY). We used Graph Pad Prism 5.00 (Graph Pad Software, San Diego, CA) to perform the statistical graphs.

## 3 Results

### 3.1 Clinical and laboratory features

Thirty-four of 179 patients died including 16 males and 18 females with no difference in sex. The median age of the fatal cases was significantly higher than that of the nonfatal cases (63 vs 57 years, respectively; *P* = 0.002). The case distribution of seasons and mortality among different age groups were shown in Supplementary Figure 1. The most frequently observed symptoms and laboratory parameters on admission are shown in Tables 1 and 2. Among these commonly presented symptoms, respiratory (55.9% vs 18.6%) and neurologic symptoms (85.3% vs 24.1%) were significantly overrepresented in fatal cases. In comparison with patients with SFTS who survived, the levels of the platelet counts, monocyte percentage, and lymphocyte percentage were identified to be significantly lower in deceased patients, whereas the viral load, neutrophil percentage, aspartate aminotransferase (AST), alanine aminotransferase (ALT), alkaline phosphatase (ALP), gamma-glutamyl transpeptidase (GGT), creatinine (Cr), lactate dehydrogenase (LDH) and creatinine kinase (CK) values were significantly higher, and activated partial thromboplastin time (APTT), prothrombin time (PT), and thrombin time (TT) were markedly longer in deceased cases.

**Table 1:**
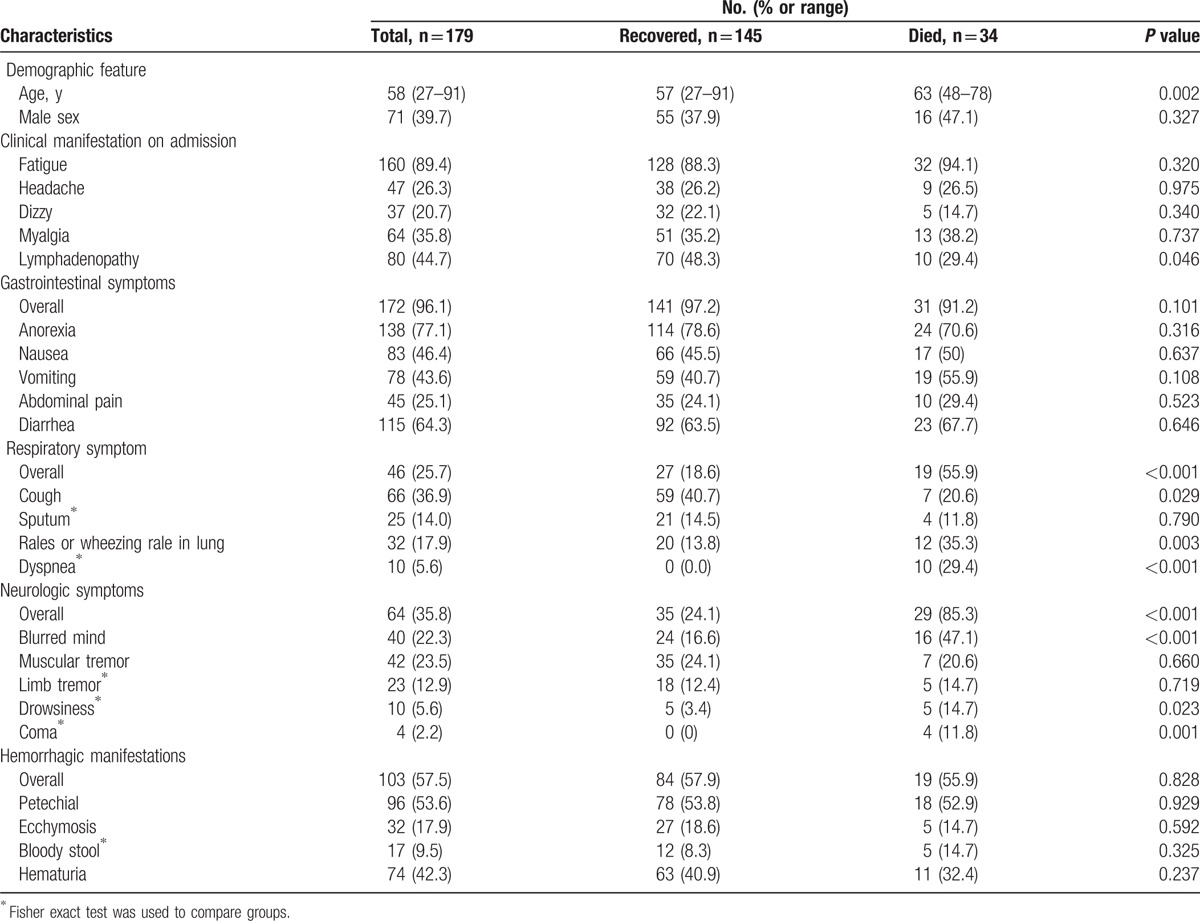
Clinical characteristics of hospitalized case-patients with confirmed severe fever with thrombocytopenia syndrome.

**Table 2:**
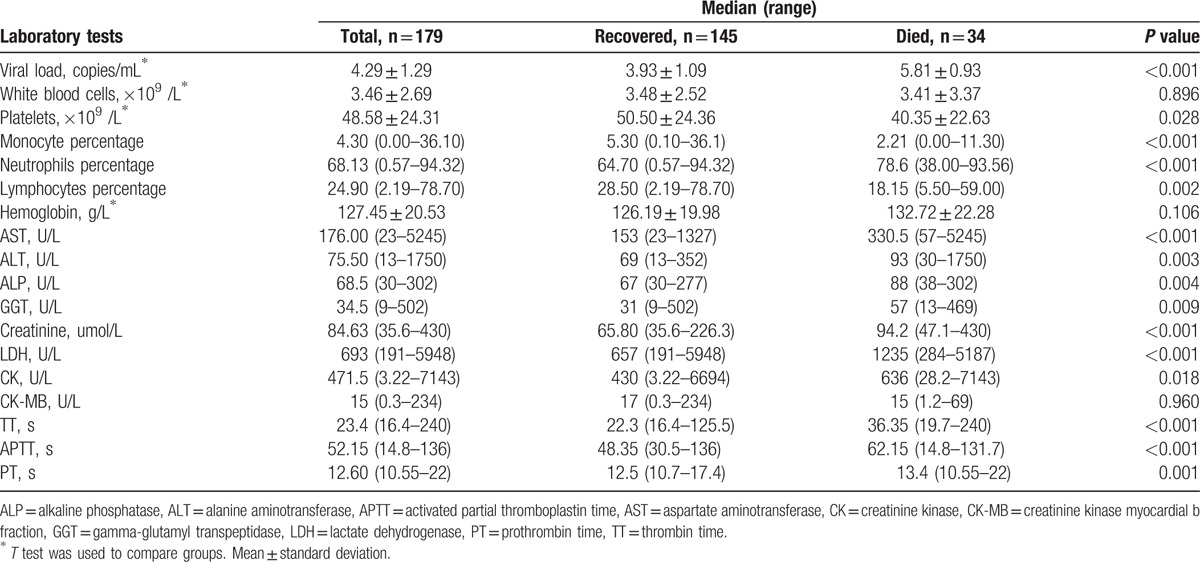
Laboratory features of hospitalized case-patients with confirmed severe fever with thrombocytopenia syndrome by outcome on admission.

### 3.2 Risk factors for mortality

Univariate regression analyses revealed that older age (OR, 1.068; 95% CI, 1.024–1.113; *P* = 0.002), increased level of neurologic symptoms (OR, 6.995; 95% CI, 3.593–13.618; *P* < 0.001), respiratory symptoms (OR, 4.192; 95% CI, 2.375–7.398; *P* <0.001), viral load (OR, 5.954; 95% CI, 3.194–11.099; *P* <0.001), alanine aminotransferase (OR, 1.932; 95% CI, 1.316–2.837; *P* = 0.001), aspartate aminotransferase (OR, 1.689; 95% CI, 1.281–2.227; *P* < 0.001), gamma-glutamyl transpeptidase (OR, 1.766; 95% CI, 1.128–2.765; *P* = 0.01), creatinine (OR, 3.518; 95% CI, 1.860–6.656; *P* <0.001), lactate dehydrogenase (OR, 2.220; 95% CI, 1.487–3.314; *P* <0.001), creatinine kinase (OR, 1.344; 95% CI, 0.993–1.820; *P* = 0.06), thrombin time (OR, 2.828; 95% CI, 1.703–4.698; *P* = 0.001), the activated partial thromboplastin time (OR, 4.533; 95% CI, 1.881–10.926; *P* <0.001), and decreased level of monocyte percentage (OR, 0.411; 95% CI, 0.272–0.621; *P* <0.001) were the independent risk factors for fatal outcomes (Table 3). Multivariate regression analyses indicated that elevated levels of neurologic symptoms (OR, 6.068; 95% CI, 2.076–17.730; *P* = 0.001), respiratory symptoms (OR, 4.480; 95% CI, 1.654–12.134; *P* = 0.003), viral load (OR, 5.017; 95% CI, 1.868–13.478; *P* = 0.001), and a lower level of monocyte percentage (OR, 0.347; 95% CI, 0.156–0.768; *P* = 0.01 were the critical risk factors for fatal outcomes (Table 4).

**Table 3:**
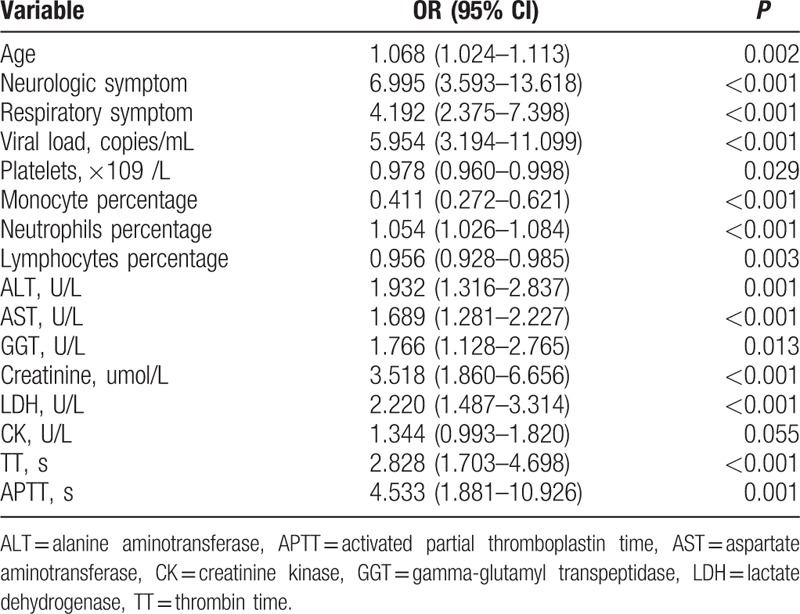
Univariate logistic regression analysis of variables associated with fatal outcome.

**Table 4:**
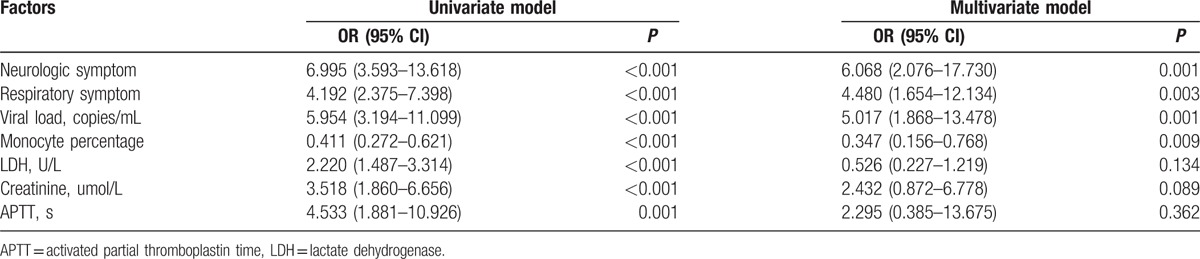
Univariate and multivariate analyses of features associated with mortality in SFTS patients.

### 3.3 Clinical scoring model proposed for predicting SFTS mortality

To access the contribution of these variables to mortality on admission, we analyzed the variables of the viral load, neurologic symptoms, respiratory symptoms, and monocyte percentage in multiple linear regression analyses. We found that a higher viral load, neurologic symptoms, respiratory symptoms levels, and lower monocyte percentage significantly affected the hospital fatality rate (*P* <0.001, <0.001, <0.001, and = 0.002, respectively; Table 5). A simple and practical clinical scoring model, the SFTS index (SFTSI), to predict the hospital mortality on admission after infected SFTSV was established. This index was calculated using the viral load, monocyte percentage, and levels of neurologic and respiratory symptoms: SFTSI = 5 × Neurologic symptoms-level + 4 × Respiratory symptoms-level + 3 × LG10 Viral load – 2 × LN Monocyte% – 7.

**Table 5:**
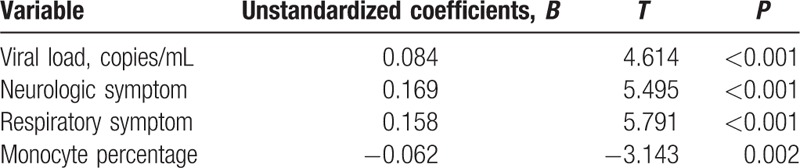
Multiple linear regression analyses to assess the contribution of variables to mortality.

### 3.4 Validation of the score model

**Figure 1:**
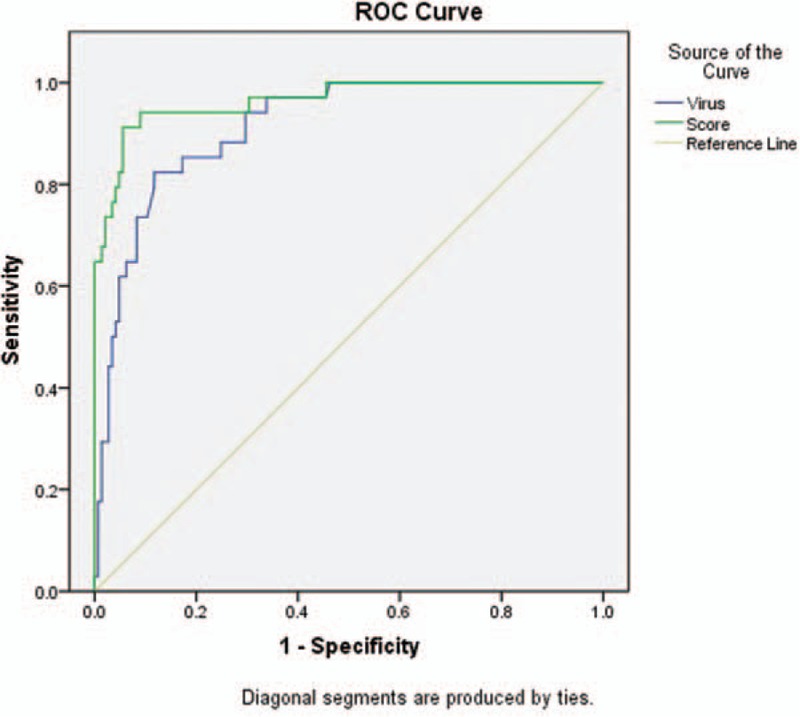
ROC curves for SFTS index and viral load in SFTS patients. ROC = receiver operating characteristic, SFTS = severe fever with thrombocytopenia syndrome.

**Figure 2:**
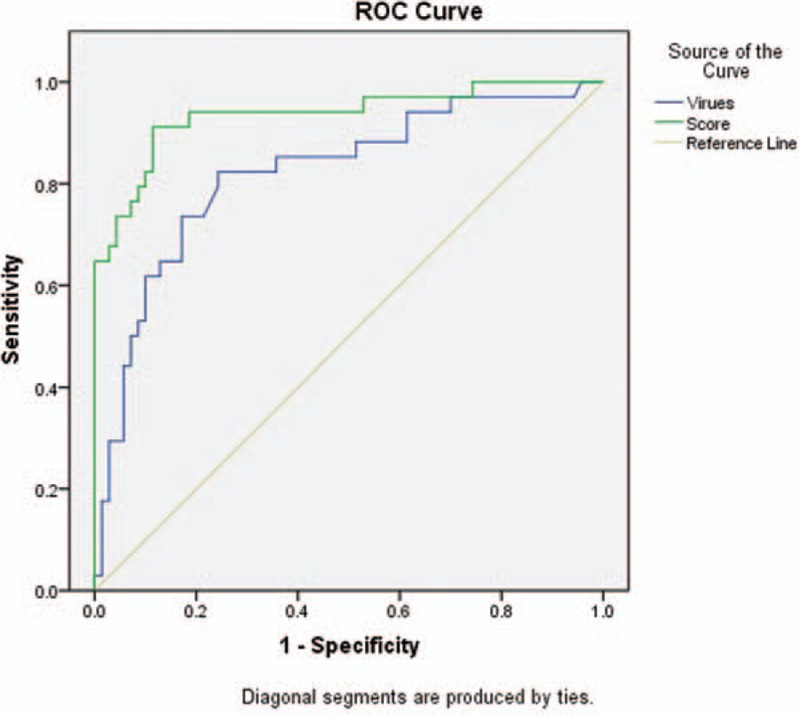
ROC curves for SFTS index and viral load in patients with viral load > 10^4^ copies/mL. ROC = receiver operating characteristic, SFTS = severe fever with thrombocytopenia syndrome.

**Figure 3:**
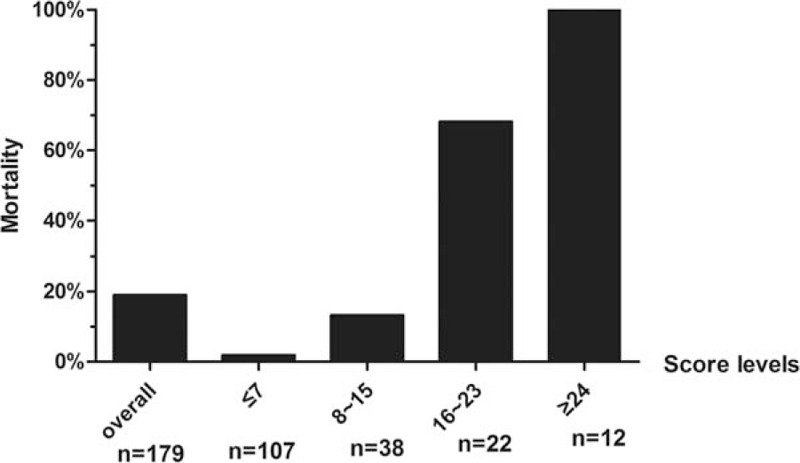
Hospital mortality increases as the SFTS index level increases. SFTS = severe fever with thrombocytopenia syndrome.

ROC analyses were performed to evaluate the predictive value of the SFTSI (Fig. 1). The SFTSI for predicting the mortality after infection with SFTSV showed an AURC of 0.965 (95% CI: 0.932–0.997, *P* <0.001), which is higher than viral load alone at 0.913 (95% CI: 0.867–0.960, *P* <0.001). Because the viral load of all the deceased cases were above 10^4^ copies/mL (Supplementary Table 2), the AURCs of SFTSI and viral loads for the prediction of hospital mortality among patients with viral load >10^4^ copies/mL were further detected and showed that the AURC of SFTSI was obviously higher than the AURC of virus alone (0.936 vs 0.821) (Fig. 2). Furthermore, the SFTSI level is positively correlated with the fatality rate. The hospital mortality in different ranges of SFTSI are shown in Fig. 3. All patients with an SFTSI⩾24 died, while patients with an SFTSI≤7 (mortality 1.9%) rarely died. When the SFTSI was distributed between 16 and 23, the mortality was obviously higher than the overall mortality (68.2% vs 19.0%, respectively). Compared with the overall mortality, there was a slight difference in the mortality of patients with SFTS from 8 to 15 (13.2% vs 19.0%, respectively). These findings might suggest that the best cut-off value of SFTSI for the fatal rate was 16; the sensitivity, specificity, and Youden index were 0.77, 0.97, and 0.73, respectively. The patients with SFTSIs higher than 16 were much more likely to die after being infected with SFTSV.

## 4 Discussion

The aim of this study was to establish a scoring system for predicting the prognosis and assessing the severity of SFTS patients. After identifying the 4 critical risk factors of viral load, monocyte percentage, respiratory, and neurologic symptoms for mortality, we proposed the following scoring formula: SFTSI = 5 × Neurologic symptoms-level + 4 × Respiratory symptoms-level + 3 × LG10 Viral load – 2 × LN Monocyte% – 7. This formula provides a simple and practical method for clinicians to evaluate the outcomes of SFTS patients on admission.

Previous studies also identified several risk factors for fatal outcomes. A higher serum viral load; older age; decreased white blood cell counts, platelet counts, lymphocyte percentage, and albumin; and an elevated neutrophil percentage, AST, ALT, LDH, CK, ALP, GGT, BUN, and CREA were identified to be risk factors for death.^[5–8]^ In addition, patients with acute lung injury/acute respiratory distress syndrome, central nervous system (CNS) symptoms, hemorrhagic manifestations, and disseminated intravascular coagulation are more likely to die.^[9–11]^ The majority of these results are consistent with our findings by univariate regression analysis. A unique feature of this study was the finding that only 2 clinical symptoms (neurologic and respiratory symptoms) and 2 laboratory parameters (viral load and monocyte percentage) were critical risk factors for fatal outcomes by multiple regression analyses.

SFTSV infection could cause MOF by releasing the proinflammatory factors including acute phase proteins (phospholipase A, fibrinogen, hepcidin), cytokines (IL-6, IL-8, IL-10, IL-1RA, IL-1β, interferon-γ, TNF- a, IFN-γ, G-CSF, and MCP-1, MIP-1α, and MIP-1β), and chemokines (IL-8, monocyte chemotactic protein 1, macrophage inflammatory protein 1b, IP-10).^[7,12,13]^ It was also observed that most serious patients would develop multiple organ dysfunction syndrome (MODS), which was significantly associated with death, and the cumulative Marshall score was significantly higher in the death group than that in the survival group.^[14]^ The commonly used formulas (Supplementary Table 3) for evaluating the severity of patients with MODS, such as Marshall, LODS, SOFA, APACHE II, REMS, and MEWS etc., were not suitable for SFTS because of the unique characteristics of SFTS. First, all SFTS patients have a lower platelet counts. Second, although some patients have liver, heart and renal dysfunction, these features were not critical factors for predicting the outcomes of SFTS patients. Third, this is an acute infectious disease, and most patients are farmers who had no chronic diseases. Furthermore, examinations for PaO2/FiO2 and PAR are seldom available for primary care physicians in the countryside, who are likely the first group of doctors to identify these diseases. In contrast, viral load as a key factor to predict the outcomes of SFTS is not included in these traditional MODS scoring systems. In addition to SFTS virus, we found that the monocyte percentage is another critical factor in predicting the outcomes, which is in line with a previous study showing SFTS fatal cases with decreased monocyte cell counts and subsets.^[15]^ Therefore, the scoring system established for SFTS in this study is not only very specific, but it is also easy to be applied in the undeveloped SFTSV epidemic areas. Furthermore, compared with the sole factor of viral load for predicting the outcomes, SFTSI had a higher accuracy, especially among patients with a high viral load.

We are undertaking a prospective study on a larger cohort of SFTS patients hospitalized in our hospital in 2016 to validate the predictive value of this model. Furthermore, the performance of this scoring system will be validated on external patients and the formula will be updated based on the validation results. Our study developed a simple and practical score formula to predict the outcomes of SFTS patients. This model provides a valuable method for clinicians to stratify the patients quickly and then provide prompt supportive therapy.

## Acknowledgments

The authors gratefully acknowledge the study subjects, clinical sites, Department of Infectious Diseases, Union Hospital of Tongji Medical College, Huazhong University of Science and Technology, and Ting-ting Qing and Yuan-li Chen for their providing statistic consultations.

## Supplementary Material

Supplemental Digital Content
