# Supplementary material for: A simple and practical score model for predicting the mortality of severe fever with thrombocytopenia syndrome patients
Source: Medicine (Baltimore). 2016 Dec 30;95(52):e5708. doi: 10.1097/MD.0000000000005708 (PMC5207567; doi:10.1097/MD.0000000000005708)
Supplement: Supplemental Digital Content [file medi-95-e5708-s001.doc]

**Supplementary Figure 1**. Epidemic curve for severe fever with thrombocytopenia syndrome cases diagnosed and mortality among different age groups during 2015. **A)** Monthly hospital admissions of patients with severe fever with thrombocytopenia syndrome virus infection. **B)** Age distribution of the severe fever with thrombocytopenia syndrome mortality.


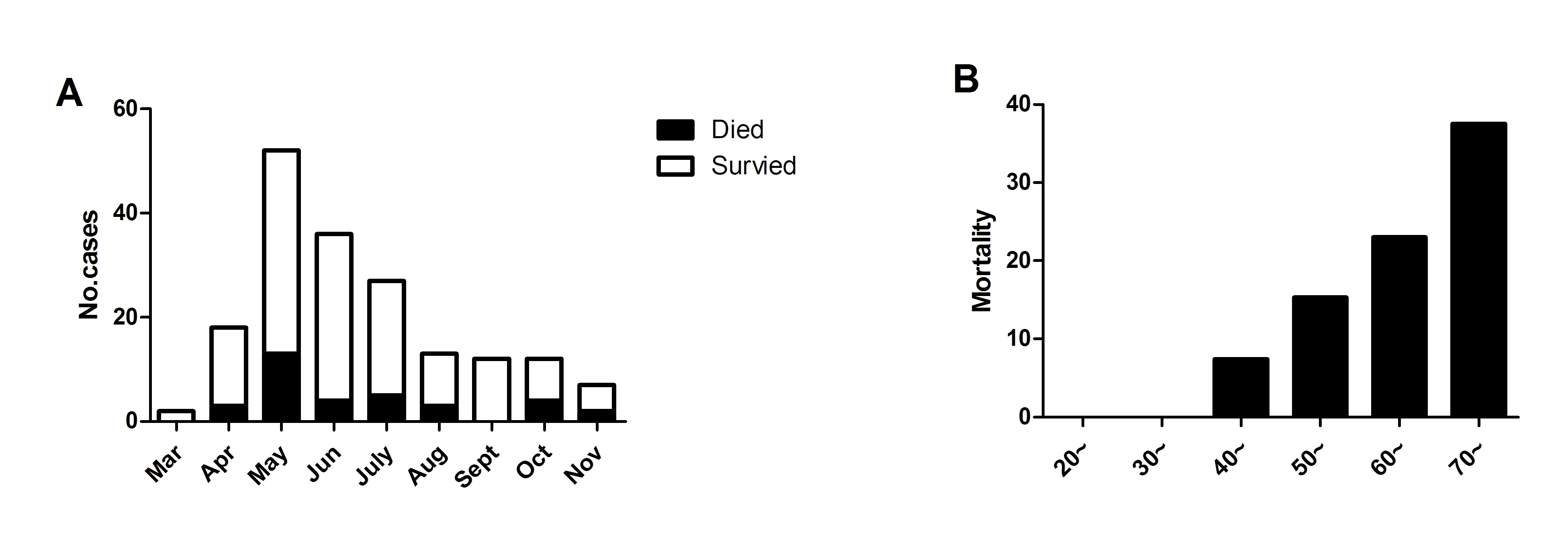


**Supplementary Table 1.** Scoring methods of respiratory and neurologic symptoms.

| System level | 0 | 1 | 2 | 3 |
| --- | --- | --- | --- | --- |
| Respiratory  symptoms | no referred symptoms | cough and sputum or appearance of pulmonary rales in lung | sputum and appearance of pulmonary rales or wheezing rale in lung | dyspnea or extensive pulmonary rales or wheezing rale in lung |
| Neurologic symptoms | no referred symptoms | limb tremor/blurred mind/slower reaction | strong decrease consist of drowsiness or frequent muscle convulsion | serious decrease of coma |

**Supplementary Table 2.** Cases distribution among different level virus load.

| LG Viral load, copies/mL | Dead cases | Alive cases |
| --- | --- | --- |
| >7 | 4 | 1 |
| 6~7 | 9 | 3 |
| 5~6 | 15 | 13 |
| 4~5 | 6 | 48 |
| <4 | 0 | 80 |

**Supplementary Table 3.** Commonly used scoring methods for patients.

| Organ system | Respiratory | Renal | Hepatic | Cardiovascular | CNS^1^ | Hematologic | | Others |
| --- | --- | --- | --- | --- | --- | --- | --- | --- |
| MODS score | PaO_2_/FiO_2_ | Creatinine | Total bilirubin | PAR^2^ | Glasgow coma scale | | Platelet | NA^4^ |
| SOFA | PaO2/FiO2 | Creatinine | Total bilirubin | Hypotension | Glasgow coma scale | | Platelet | Urine volume |
| LODS | PaO_2_/FiO_2_ | Creatinine | Total bilirubin | PAR | Glasgow coma scale | | Platelet | NA |
| REMS | Respiratory rate, oxyhemoglobin saturation | NA | NA | Systolic blood pressure, pulse | Glasgow coma scale | | NA | age |
| MEWS | Respiratory rate,  oxyhemoglobin saturation | NA | NA | Heart rate, Systolic blood pressure | Consciousness | | NA | temperature |
| APACHEⅡ^3^ | Respiratory rate,  oxygenation index | Creatinine | NA | Mean blood pressure, Heart rate | Glasgow coma scale | | NA | Age, pH temperature |

^1^CNS: central nervous system.

^2^PAR=heart rate ×central venous pressure/mean arterial blood pressure.

^3^APACHE Ⅱ consists of age, acute physiology and chronic health evaluation and there some other common test items that are not listed in above table.

^4^NA: not applicable.
